# Non-Invasive Radiomics Approach Predict Invasiveness of Adamantinomatous Craniopharyngioma Before Surgery

**DOI:** 10.3389/fonc.2020.599888

**Published:** 2021-02-17

**Authors:** Guofo Ma, Jie Kang, Ning Qiao, Bochao Zhang, Xuzhu Chen, Guilin Li, Zhixian Gao, Songbai Gui

**Affiliations:** ^1^ Department of Neurosurgery, Beijing Tiantan Hospital, Capital Medical University, Beijing, China; ^2^ Department of Radiology, Beijing Tiantan Hospital, Capital Medical University, Beijing, China; ^3^ Neuropathology Department, Beijing Neurosurgical Institute, Capital Medical University, Beijing, China

**Keywords:** craniopharyngioma, adamantinomatous, invasiveness, radiomics, machine learning, nomogram

## Abstract

**Purpose:**

Craniopharyngiomas (CPs) are benign tumors, complete tumor resection is considered to be the optimal treatment. However, although histologically benign, the local invasiveness of CPs commonly contributes to incomplete resection and a poor prognosis. At present, some advocate less aggressive surgery combined with radiotherapy as a more reasonable and effective means of protecting hypothalamus function and preventing recurrence in patients with tight tumor adhesion to the hypothalamus. Hence, if a method can be developed to predict the invasiveness of CP preoperatively, it will help in the development of a more personalized surgical strategy. The aim of the study was to report a radiomics-clinical nomogram for the individualized preoperative prediction of the invasiveness of adamantinomatous CP (ACPs) before surgery.

**Methods:**

In total, 1,874 radiomics features were extracted from whole tumors on contrast-enhanced T1-weighted images. A support vector machine trained a predictive model that was validated using receiver operating characteristic (ROC) analysis on an independent test set. Moreover, a nomogram was constructed incorporating clinical characteristics and the radiomics signature for individual prediction.

**Results:**

Eleven features associated with the invasiveness of ACPs were selected by using the least absolute shrinkage and selection operator (LASSO) method. These features yielded area under the curve (AUC) values of 79.09 and 73.5% for the training and test sets, respectively. The nomogram incorporating peritumoral edema and the radiomics signature yielded good calibration in the training and test sets with the AUCs of 84.79 and 76.48%, respectively.

**Conclusion:**

The developed model yields good performance, indicating that the invasiveness of APCs can be predicted using noninvasive radiological data. This reliable, noninvasive tool can help clinical decision making and improve patient prognosis.

## Introduction

Craniopharyngiomas (CPs) are rare and non-neuroepithelial entities arising from a malformation of embryonal tissue, with an incidence of 0.5–2 cases per million persons per year (1–3). Two histological subtypes have been identified: adamantinomatous CPs (ACPs) and papillary CPs (PCPs). They are commonly located in the suprasellar region and can cause devastating neuroendocrine dysfunction by mass effect and/or invasion to the optic apparatus, pituitary gland and hypothalamus. Complete tumor resection with improvement in visual function, and no further deterioration of neuroendocrine and cognitive function is considered the optimal treatment outcome. However, although these massed are of a benign histological nature, the abovementioned ideal treatment goal is not always achievable due to the potential close adhesion of CPs to surrounding brain tissue.

Pathological studies have confirmed that the histology of the interface between CPs and surrounding brain tissue can be classified into two types, including finger-like invasion and no finger-like invasion (4–6). Numerous investigators have deemed that such local invasion resulting in adhesion could be associated with the failure of complete resection and poor prognosis (7–9). Therefore, a preoperative noninvasive method for identifying the invasiveness of CPs could help in the development of more individualized treatment decisions. Addressing this problem, we developed a machine learning radiomics model to predict the invasiveness of ACPs before surgery.

Radiomics is an emerging research method that can effectively evaluate the heterogeneity of tumors by extracting a large number of image features from medical images. Its applicability and utility have already been validated in several tumor types; Zhang et al. focused on the preoperative prediction of nonfunctioning pituitary adenoma subtypes before surgery (10); Li et al. predicted P53 status, progression-free survival (PFS), phosphatase and tensin homolog (PTEN) and vascular endothelial growth factor (VEGF) expression in patients with gliomas (11–14). Furthermore, radiomics approaches have also been validated in meningiomas (15), lung cancer (16) and skull base chordomas (17).

In the current study, we extracted a large number of radiomics features from preoperative MRI scans of ACPs with known local invasiveness. We hypothesized that a radiomics model could predict the invasiveness of ACPs via a machine-learning algorithm.

## Methods

### Patients

We retrospectively reviewed the medical records of patients who underwent surgery for craniopharyngioma from 2002 to 2019, and a total 335 cases of ACPs were included in this study. Their radiographic and pathological data were collected from picture archiving and communications systems. The pathological sections were reviewed by two individual senior neuropathologists to confirm the histology of the interface between the ACPs and surrounding brain tissue (**Figures S2 and S4**). Potential candidates were excluded if their pathological sections could not reflect the relationship between the ACP and brain tissues. Furthermore, MRI images were reviewed by two experienced radiologists to identify whether peritumor edema was present on T2-weighted images. Any disagreement was resolved by a consultation. The inclusion criteria were as follows: 1) histologically confirmed as ACPs; 2) the definite invasiveness of each tumor; 3) complete preoperative MRI data [including T2-weighted, T1-weighted and contrast enhanced (CE)-T1 images]; 4) no history of surgical treatment; and 5) available clinical characteristics. Among 335 patients, 225 patients who were treated between January 2002 and December 2015 were allocated to the training set, and 110 patients who were treated between January 2016 and December 2019 were allocated to the validating set. The training set was used to establish a stable model to predict the invasiveness of ACPs via radiomics features, while the validation set was used to assess the prediction accuracy of the model. The study was approved and reviewed by the institutional review board.

### MRI Acquisition and Tumor Segmentation

CE-T1 images were used for the extraction of radiomics features, as these images are optimal for identifying the tumor border. MRI was performed in the head-first supine position on a 3-T scanner (Tim Trio, Siemens) using a head coil. The acquisition parameters for precontrast T1-weighted sequences were as follows: repetition time, 156–2,520 ms; echo time, 2–19.7 ms; flip angle:150°; field of view: 240×188 mm^2^; acquisition matrix: 384×300 and slice thickness: 5 mm. The study was repeated immediately after the rapid injection of contrast agent gadolinium-DTPA (0.1 mmol/kg Gadovist; Beijing Beilu Pharmaceutical Co., Beijing China). The regions of interest (ROI), i.e., whole tumors, were manually delineated by two neuroradiologists on the CE-T1 images using MRIcron software (http://www.mccauslandcenter.sc.edu/mricro) (**Figure S1** and **S3**). The two neuroradiologists were blinded to the patients’ clinical characteristics. Next, a third senior neuroradiologist reevaluated the ROIs and made final decisions when discrepancies were ≥ 5%.

### Feature Extraction

First, we homogenized the image intensity on all MR images by z-score transformation (MATLAB version 2014a; The Mathworks, Natick, MA, USA) to avoid heterogeneity bias. In this study, a total of 1,874 features were acquired (**Table S1**). The features were divided into eight categories: (a) first-order statistics, (b) shape-based, (c) Gray Level Cooccurence Matrix (GLCM), (d) Gray Level Run Length Matrix (GLRLM), (e) Gray Level Size Zone Matrix (GLSZM), (f) Neighboring Gray Tone Difference Matrix (NGTDM), (g) Gray Level Dependence Matrix (GLDM), and (h) wavelet features, they were derived from first-order statistics and texture features via wavelet decomposition (using directional low-pass and high-pass filtering.

### Feature Selection and Classification

We used the least absolute shrinkage and selection operator (LASSO) algorithm, which is a suitable and powerful method for the regression of high-dimensional data, to screen the most predictive features in the training set. In this procedure, the tuning parameter (lambda) was selected by the cross-validation method; the optimal lambda was confirmed as that which resulted in the smallest cross-validation error. Then, a support vector machine (SVM) classifier was used to establish a machine-learning model for invasiveness prediction. The performance of the classification model was evaluated and validated by employing 10-fold cross-validation. Receiver operator characteristic (ROC) curve analysis was performed for both the training and validation sets to evaluate the discriminative ability of the machine-learning model.

### Radiomics-Clinical Nomogram Construction and Performance Assessment

To provide a more individualized predictive model, a nomogram was built from the training set data. First, a radiomics signature was constructed using the selected features, and represented by a radiomics score. The score was calculated for each patient as a linear combination of the selected features weighted by their respective coefficients. Second, the radiomics signature and other clinical predictors (age, sex, peritumoral edema, tumor size) were tested using a multivariate logistic regression algorithm in the training set. The final selection of the model for the nomogram was conducted using a backward step-down selection process based on the Akaike information criterion. The performance of the nomogram was estimated with the training cohort and then tested with the validation cohort.

### Statistics

The Mann–Whitney U test and chi-square test were used to evaluate whether age, sex, tumor invasiveness and peritumoral edema were significantly different between the training set and validation set. They were performed by using SPSS software version 22.0 (IBM Corp.) Statistical significance was set as p < 0.05. The LASSO algorithm, SVM classifier, ROC curve analysis and nomogram were performed based on “glmnet”, “e1071”, “pROC”, and “rms” packages in R software version 3.3.2 (The R Foundation, Salt Lake City, UT, USA), respectively.

## Results

### Clinical Characteristics

A total 187 male and 148 female patients was enrolled in the study, with 51 pediatric patients (mean age 14.3 years, range 6–17 years) and 284 adult patients (mean age 41.6 years, range 18–71 years). Among these patients, 129 men and 96 women were allocated to the training group and 58 men and 52 women were allocated to the validation group via random assignment. The ratios of invasiveness to noninvasiveness were 65/160 in the training group and 31/79 in the validation set. The distributions of the characteristics of the two groups were compared using the Mann–Whitney U test and the chi-square test, and there were no significant differences in age (p = 0.61), sex (p = 0.43), peritumoral edema (p = 0.38), tumor size or invasiveness (p = 0.22). Detailed information pertaining to the clinical characteristics of the patients is shown in **Table 1**.

**Table 1:** Patient characteristics.

	Training	Validation	P value
Age(years, mean)	37.2	35.63	0.61^a^
Sex(Male/Female)	129/96	58/52	0.43^b^
Peritumoral edema	48/177	19/91	0.38^b^
Tumor invasiveness	65/120	31/79	0.22^b^

^a^Mann-Whitney U test, ^b^Chi-square test.

### Machine-Learning Model for Predicting the Invasiveness of ACPs

**Figure 1:**
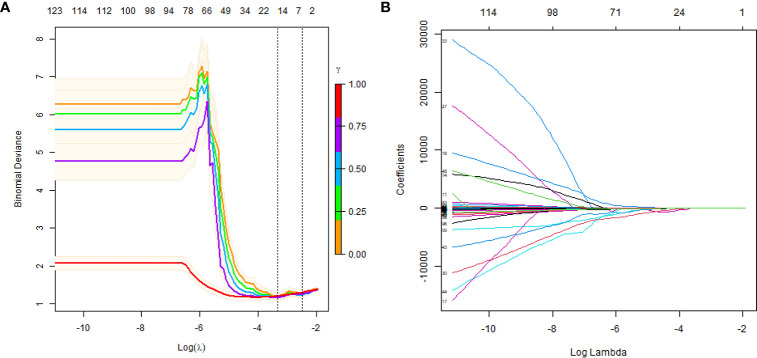
Texture feature selection using LASSO logistic regression. **(A)** Selection of the tuning parameter (lambda). The dotted vertical lines are plotted at the optimal λ values based on the minimum criteria and 1 standard error of the minimum criteria. **(B)** LASSO coefficient profiles are shown for the 1874 texture features. A vertical line is drawn at the value where the optimal lambda results in 11 nonzero coefficients.

In this study, the LASSO algorithm was used to select features with nonzero coefficients, and a subset of 11 features were screened from a total of 1,874 radiomic features (**Figures 1A, B**). The names and descriptions of these 11 selected features are shown in **Table 2**.

**Table 2:** Eleven prognostic radiomics features selected by the LASSO algorithm.

Features	Descriptions	Coefficients
First order_ Skewness	Skewness measures the asymmetry of the distribution of values about the Mean value.	-2.7 × 10^-1^
GLSZM_ Gray Level Variance	Measuring the variance in gray level intensities for the zones.	4.32 × 10^-1^
Shape _Sphericity	Measuring the roundness of the shape of the tumor region relative to a circle	1.13 × 10^-1^
Shape _ Surface Volume Ratio	A lower value indicates a more compact (sphere-like) shape and dependent on the volume of the ROI.	2.46 × 10^-2^
GLCM _ Contrast	Measuring the local intensity variation, favoring values away from the diagonal.	2.87 × 10^-3^
wavelet-HLL_ GLDM_ DNU	Describing the homogeneity among dependencies in the image. The value is low if the image has more similarity.	-5.1 × 10^-1^
wavelet-LHL_GLCM_ Autocorrelation	Describing the magnitude of the fineness and coarseness of texture.	-6.72 × 10^-2^
wavelet-HLH_ NGTDM_ Busyness	Describing the change from a pixel to its neighbor. The value is high if the changes of intensity between pixels and its neighborhood is rapid.	-1.91
wavelet-LLL_ NGTDM _Complexity	Describing the complexity of the image. The value is high if there are many rapid changes in gray level intensity.	4.09 × 10^-3^
wavelet-HLL_ GLSZM_ SAHGLE	Describing the distribution of smaller size zones with higher gray-level values.	-1.17 × 10^-1^
wavelet-LLH_ GLSZM _ SZNUN	Describing the variability of size zone volumes throughout the image.	-7.42 × 10^-2^

DNU, Dependence Non Uniformity; SAHGLE, Small Area High Gray Level Emphasis; SZNUN, Size Zone Non Uniformity Normalized.

**Figure 2:**
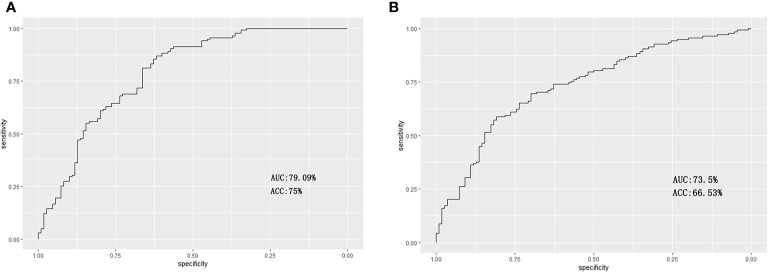
Receiver operating characteristic curves for the prediction of invasiveness of ACPs in the training and validation sets. **(A)** For the training set, the area under the curve (AUC) was 79.09% with a sensitivity, specificity and accuracy of 81.97, 66.74, and 75%, respectively. **(B)** For the validation set, the AUC was 73.5% with a sensitivity, specificity and accuracy of 69.53, 72.44, and 66.53%, respectively.

A machine-learning model was constructed based on the selected features and the SVM classifier with the training set data. The AUC was 79.09% following ROC curve analysis, and the sensitivity, specificity, and accuracy were 81.97%, 66.74 and 75%, respectively at the optimal cutoff point (0.609) (**Figure 2A**). Then, the model was applied to the validation set, and the invasiveness of the ACPs was effectively predicted. In the ROC curve analysis, the AUC was 73.5%. In addition, the optimal cutoff value (0.568) yielded a sensitivity, specificity, and accuracy of 69.53, 72.44, and 66.53%, respectively (**Figure 2B**). Hence, the 11 radiological features that constituted our model were regarded as an effective radiomics signature for the invasiveness of ACPs.

### Development and Validation of the Individualized Predictive Nomogram

**Figure 3:**
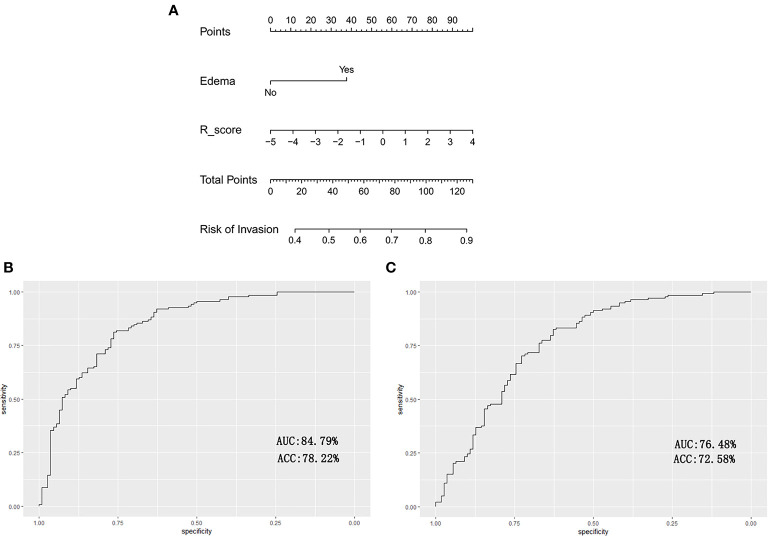
The radiomic-clinical nomogram and its performance are illustrated. **(A)** The radiomics-clinical nomogram developed to predict the invasiveness of ACPs is illustrated. **(B)** For the training set, the AUC was 84.79% with the sensitivity, specificity and accuracy of 83.27, 76.05, and 78.22%, respectively. **(C)** For the validation set, the AUC was 76.48% with a sensitivity, specificity and accuracy of 71.24, 72.33, and 72.58%, respectively.

The radiomics signature and peritumoral edema were identified as independent predictors of ACP invasiveness based on the multivariate logistic regression algorithm (**Table 3**). The nomogram showed favorable discrimination with an AUC of 84.79% [95% confidence interval (CI), 84.12–85.46%] in the training set (**Figures 3A, B**). The radiomic nomogram also showed good discrimination with an AUC of 76.48% (95% CI, 74.13–78.83%) in the testing set (**Figure 3C**).

**Table 3:** Multivariate logistic regression analysis of the radiomics score and clinical predictors in the training set.

	Univariate logistic regression			Multivariate logistic regression		
	HR	95% CI	P value	HR	95% CI	P value
Age, per 1 year increase	0.927	0.552–1.556	0.77			
Sex (male)	0.988	0.972–1.005	0.16			
Peritumoral edema	2.499	1.351–4.624	0.014	1.964	1.018–3.788	0.036
Tumor size, per 1cm increase	1.719	1.899–3.288	0.101			
Radiomics score, per 0.1 increase	1.583	1.079–2.458	<0.001	1.257	1.072–1.473	0.005

## Discussion

Although the history of surgical treatment for CPs has been spanned the course of more than 100 years, these masses still pose a surgical challenge even after the application of modern neurosurgical techniques (18–22). Numerous studies have revealed that quality of life (QoL) and cognitive performance are frequently impaired in long-term survivors after surgery due to the anatomical proximity of the CPs to the optic nerve and to the hypothalamic-pituitary axes (23–25). Some researchers have advocated less aggressive surgery combined with radiotherapy as a more reasonable and effective means of protecting hypothalamus function and preventing recurrence in the patients with tight tumor adhesion to the hypothalamus (26–28). Therefore, it is important to assess the aggressiveness of the tumor before surgery. In the present study, we used the noninvasive radiomics method to predict the invasiveness of ACPs before surgery, which made it possible to develop a personalized surgical protocol. Note that to avoid heterogeneity between the two histopathological CP subtypes, only ACPs were included in the study.

In our cohort, there were more male patients (n = 187, 55.82%) than female patients (n = 148, 44.18%), consistent with previous reports (29–31). Although CPs were more common among child patients, the proportion of adult patients was higher in this study (84.78 vs. 15.22%); because adult patients were the main group of patients in our ward.

To date, radiomics studies on craniopharyngiomas are rare. Yue et al. proposed a machine learning model for discriminating BRAF mutation and wild type among craniopharyngiomas with sensitivity of 1.00 and specificity of 0.91 (32). Chen et al. predicted the pathological subtype and gene mutations in craniopharyngiomas with radiomics (33).

Radiomics is an emerging diagnostic technique, and the potential ability of improving clinical decision support systems has been well verified. Some successful precedents have been demonstrated in radiomics studies for identifying the invasiveness of tumors. For example, a previous report showed that preinvasive pulmonary adenocarcinomas and invasive pulmonary adenocarcinomas could be distinguished by constructing a radiomics-clinical nomogram predictive model with an AUC of 0.903 (34). Another report revealed that the muscular invasiveness of bladder cancer could be evaluated by a noninvasive radiomics model (35). Furthermore, Zhu et al. proposed a learning radiomics model for preoperative grading in meningioma (36). In the present study, we employed a radiomics approach to provide preoperatively predict the invasiveness of ACPs. The high-throughput features applied in our radiomics model were extracted from the whole tumor on preoperative CE-T1 images, which could reflect the heterogeneity of the tumor. Subsequently, 11 invasiveness-associated features were screened by using the LASSO algorithm, consisting of one first-order feature, two shape-based features, two texture features, and six wavelet features. Most of these selected features were also reported in previous studies of tumor invasiveness (35, 37). Our predicted model constructed by using an SVM classifier achieved AUCs of 79.09% in the training set data and 73.5% in the validation set data. The results indicate that the invasiveness of APCs can be predicted using noninvasive radiological data, and the proposed radiomics signature performed well in the training and validation sets.

Tumor invasiveness is closely associated with gene mutations and/or relative protein expression levels. However, owing to its rarity and benign histological nature, studies of the genomics and molecular pathology of CPs are limited. A previous study revealed that the expression of claudin-1, a tight junction protein expressed in epithelial tissues that plays important roles in cell polarity and adhesion, could be strongly associated with the invasiveness of CPs (38). The authors found that the invasive CPs exhibited significantly lower claudin-1 expression than their noninvasive counterparts regardless of CP subtype. We suggest that this difference may be the basis of the molecular pathology for distinguishing invasive and noninvasive ACPs by using the radiomics method.

The individualized predictive nomogram, incorporated the radiomics signature and peritumoral edema into a model, which facilitated the individualized prediction of the invasiveness of ACPs. The radiomic-clinical nomogram showed better discrimination in the training and validation sets with AUCs of 84.79 and76.48%, respectively. This revealed that combining multiple clinical risk factors to estimate and determine follow-up treatment, rather than focusing on a single radiological feature, is very necessary.

There are some limitations in our study. First, to build the radiomics signature and predictive model, we analyzed axial CE-T1 images, which are usually referred to clinically; however, combinations with other sequences such as fluid attenuated inversion recovery (FLAIR) imaging and T2-weighted imaging may have provided additional information and improved the performance of the predictive model. Second, potential selection biases might have occurred because of the retrospective nature of the study. Third, the imaging protocols used were not fully consistent in that the imaging data were acquired with different MRI scanners.

## Conclusion

We proposed a radiomics-clinical nomogram for the individualized preoperative prediction of the invasiveness of ACPs. This reliable, noninvasive tool can help clinical decision making and improve patient prognosis.

## Data Availability Statement

The raw data supporting the conclusions of this article will be made available by the authors, without undue reservation.

## Ethics Statement

The studies involving human participants were reviewed and approved by The Ethics Committee of Beijing Tiantan Hospital affiliated to Capital Medical University. Written informed consent to participate in this study was provided by the participants’ legal guardian/next of kin.

## Author Contributions

GM collected and analyzed clinical data, and prepared the manuscript. SG designed the trial and revised the manuscript. NQ and BZ collected clinical data. XC supervised the tumor segmentation. GL supervised the evaluation of the histology of the interface between CPs and surrounding brain tissue. All authors contributed to the article and approved the submitted version.

## Conflict of Interest

The authors declare that the research was conducted in the absence of any commercial or financial relationships that could be construed as a potential conflict of interest.
