# Supplementary material for: Non-Invasive Radiomics Approach Predict Invasiveness of Adamantinomatous Craniopharyngioma Before Surgery
Source: Front Oncol. 2021 Feb 17;10:599888. doi: 10.3389/fonc.2020.599888 (PMC7925821; doi:10.3389/fonc.2020.599888)
Supplement: Supplementary file 1 [file DataSheet_1.docx]

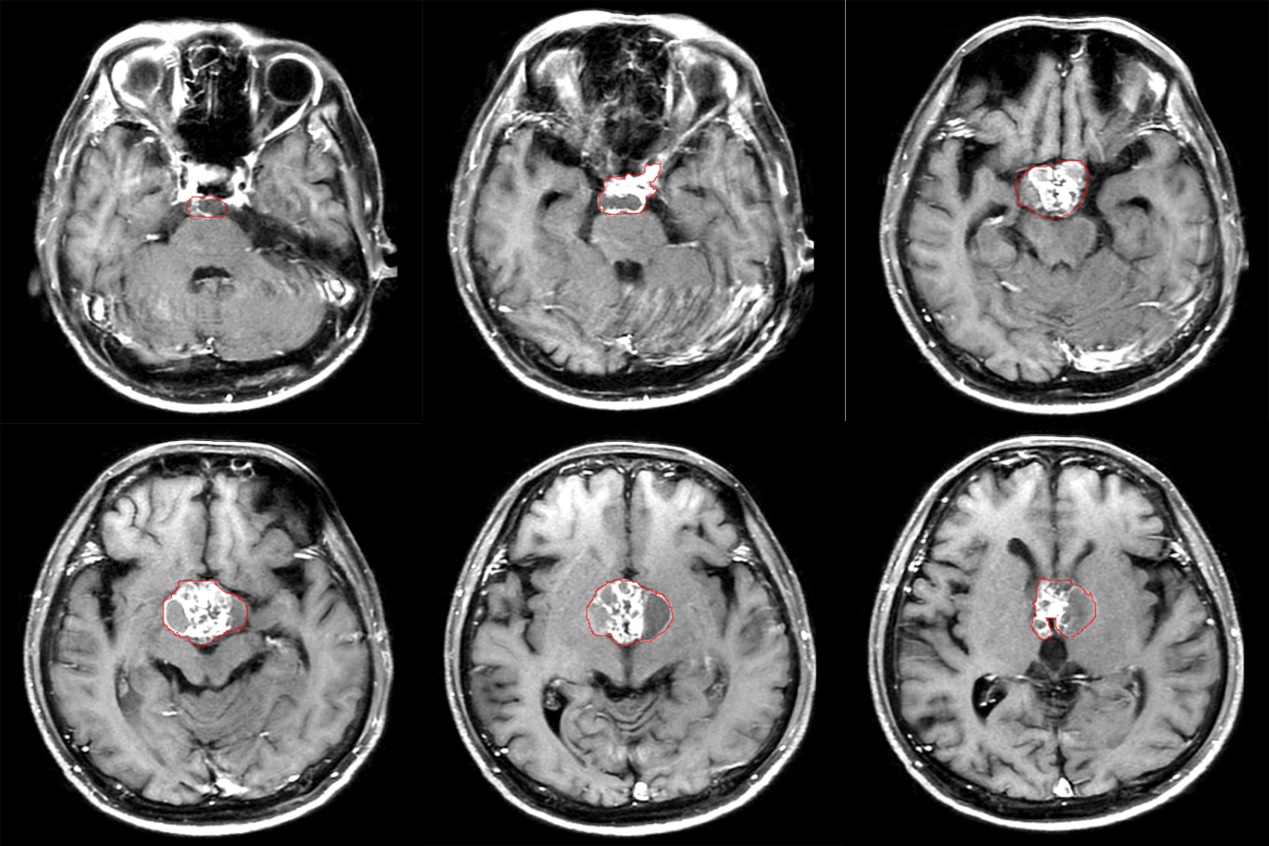


Figure S1: Tumor segmentation results of six representative axial CE-T1 images obtained from one case of ACP with the finger-like invasion between tumor and surrounding brain tissue. In each image, the area surrounded by red line indicated the tumor.


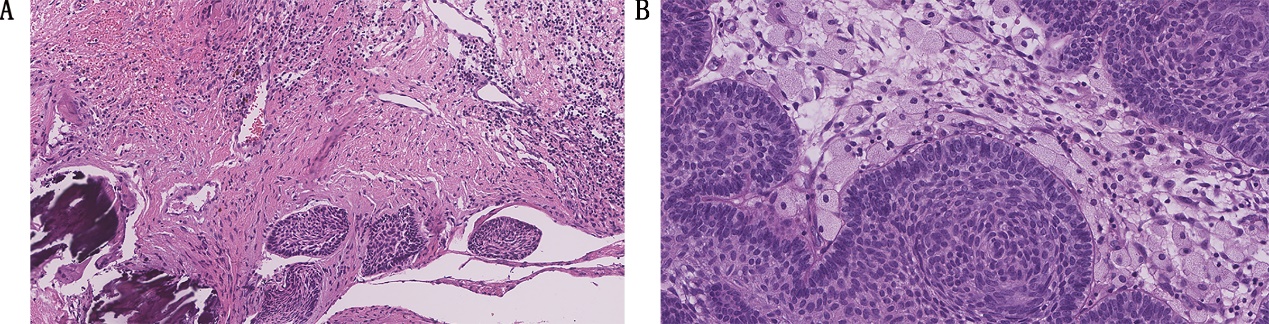


Figure S2: (A) Photomicrographs of the surgical specimens illustrating the finger-like protruding into the brain tissue. HE, original magnification ×100; (B) Photomicrographs of the surgical specimens illustrating the finger-like invasion close adhesion to the brain tissue. HE, original magnification ×400.


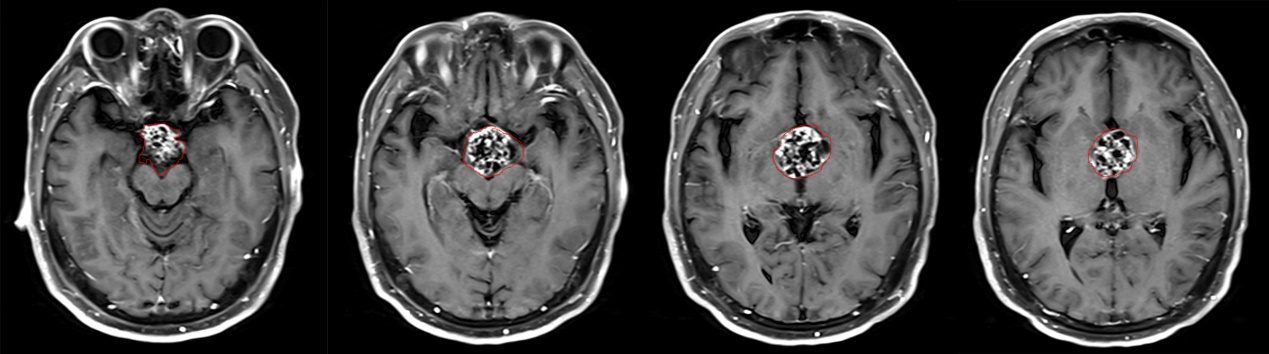


Figure S3: Tumor segmentation results of four representative axial CE-T1 images obtained from one case of ACP with no finger-like invasion between tumor and surrounding brain tissue. In each image, the area surrounded by red line indicated the tumor.


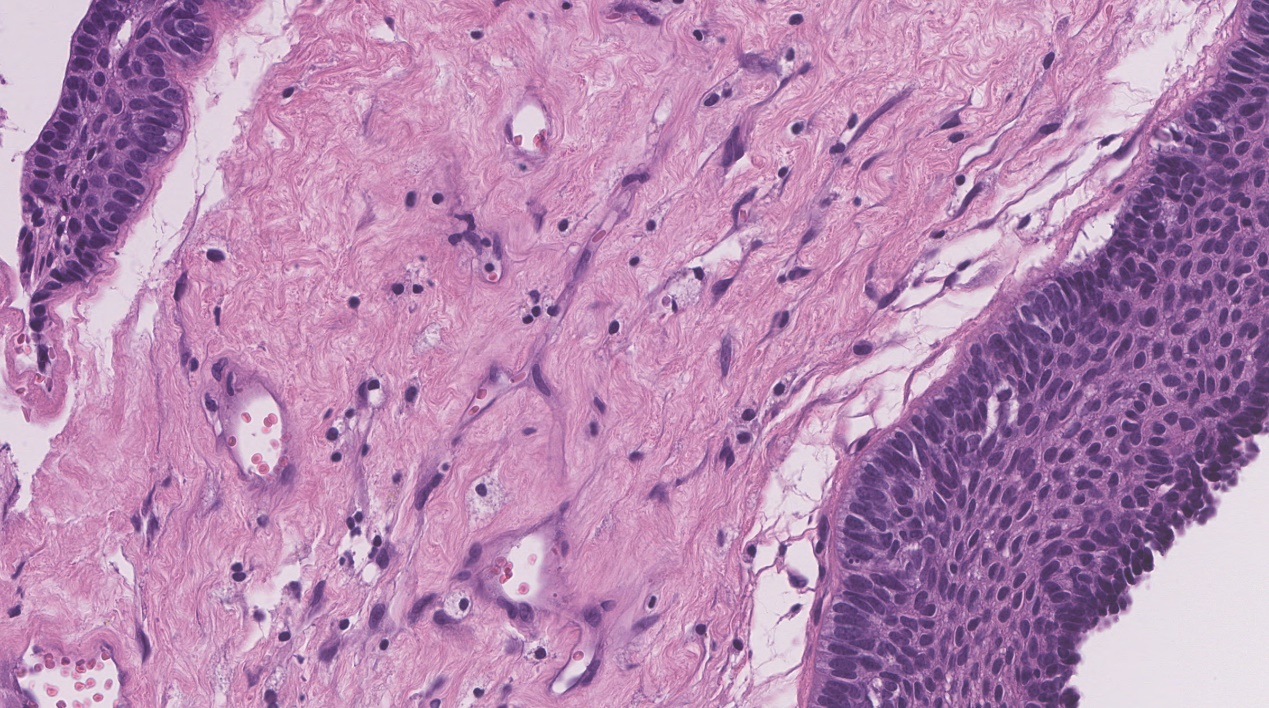


Figure S4: Photomicrographs of the surgical specimens illustrating the tumor loose adhesion to the gliosis. There are not finger-like invasion between tumor and brain tissue. HE, original magnification ×400.
