# Supplementary material for: Non-Invasive Radiomics Approach Predict Invasiveness of Adamantinomatous Craniopharyngioma Before Surgery
Source: Front Oncol. 2021 Feb 17;10:599888. doi: 10.3389/fonc.2020.599888 (PMC7925821; doi:10.3389/fonc.2020.599888)
Supplement: Supplementary file 2 [file Table_1.docx]

**Supplement Table S1** The four groups of texture features

| Group 1.  First order statistics  (n = 18*12=216) | Group 2.  Shape and size based features  (n = 14) | Group 3.  Texture features  (n = 12*24+12*16*2+12*5+12*14=900) | Group 4.  Wavelet features  (n = 18*8+75*8=744) |
| --- | --- | --- | --- |
| Energy; Entropy; Kurtosis; Maximum; Mean; Mean Absolute Deviation; Median; Minimum; Range; Root Mean Square; Skewness; Uniformity; Variance; Total energy; Robust Mean Absolute Deviation; Interquartile Range; 10Percentile; 90Percentile. | Elongation; Flatness; Least axis length; Major axis length; Maximum 2D diameter column; Maximum 2D diameter row;  Maximum 2D diameter slice;  Maximum 3D diameter; Mesh volume; Minor axis length; Sphericity; Surface area; Surface to volume ratio; Voxel volume | GLCM: Autocorrelation; Cluster prominence; Cluster shade; Cluster tendency; Contrast; Correlation; Difference entropy; Dissimilarity; Energy; Inverse variance; joint average; Maximum probability  GLRLM: Gray level nonunifomity; Gray level nonunifomity normalized; Gray level variance; High Gray level Run emphasis;…; Short run low gray level emphasis  GLSZM: Gray level nonunifomity; Large area high gray level emphasis; Size zone nonuniformity normalized;…Zone variance.  NGTDM: Busyness; Coarseness; Complexity; Contrast; Strength  GLDM: Dependence entropy; Low gray level emphasis;…; Small dependence emphasis; Small dependence low gray level emphasis. | Energy_LLL; Energy_LLH; Energy_LHL; Energy_LHH; Energy_HLL; Energy_HLH; Energy_HHL; Energy_HHH; …; Long run high gray level emphasis_LLL; Long run high gray level emphasis_LLH; Long run high gray level emphasis_LHL; Long run high gray level emphasis_LHH; Long run high gray level emphasis_HLL; Long run high gray level emphasis_HLH; Long run high gray level emphasis_HHL; Long run high gray level emphasis_HHH; |
